# The deubiquitinase USP22 regulates PD-L1 degradation in human cancer cells

**DOI:** 10.1186/s12964-020-00612-y

**Published:** 2020-07-14

**Authors:** Yu Wang, Qingguo Sun, Ning Mu, Xiaoyang Sun, Yingying Wang, Songqing Fan, Ling Su, Xiangguo Liu

**Affiliations:** 1 Shandong Provincial Key Laboratory of Animal Cell and Developmental Biology, School of Life Sciences, Shandong University, 72 Binhai Road, Qingdao, 266237 P. R. China; 2 Shandong Provincial Collaborative Innovation Center of Cell Biology, School of Life Sciences, Shandong Normal University, Jinan, China; 3 Department of Pathology, The Second Xiangya Hospital of Central South University, Changsha, China

**Keywords:** PD-L1, USP22, CSN5, Deubiquitination, Immune checkpoint blockade therapy

## Abstract

**Background:**

Many cancers evade immune surveillance by overexpressing PD-L1. PD-L1 interacted with its receptor PD-1, resulting in reduction of T cell proliferation and activation and thereafter cancer cell death mediated by T-lymphocyte. Understanding the mechanisms that regulate PD-L1 was of vital importance for immune checkpoint blockade therapy (ICBT).

**Methods:**

Human non-small cell lung cancer cells and 293FT cells were used to investigate the function of USP22 upon PD-L1 and CSN5 by WB, Immunoprecipitation, Immunofluorescence and Flow cytometry analysis. B16-F10 cells were used to explore the role of USP22 on tumorigenesis and T cell cytotoxicity. The relationship between USP22 and PD-L1 expression was investigated by Immunohistochemistry analysis in human non-small cell lung cancer samples.

**Results:**

Our data showed that USP22 interacted with PD-L1 and promoted its stability. USP22 deubiquitinated PD-L1 and inhibited its proteasome degradation. Moreover, USP22 also interacted with CSN5 and stabilized CSN5 through deubiquitination. Either USP22 or CSN5 could facilitate the interaction of PD-L1 with the other one. Furthermore, USP22 removed K6, K11, K27, K29, K33 and K63-linked ubiquitin chain of both CSN5 and PD-L1. In addition, USP22 depletion inhibited tumorigenesis and promoted T cell cytotoxicity. Besides, USP22 expression positively correlated with PD-L1 expression in human non-small cell lung cancer samples.

**Conclusions:**

Here, we suggested that USP22 is a new regulator for PD-L1. On the one hand, USP22 could directly regulate PD-L1 stability through deubiquitination. On the other hand, USP22 regulated PD-L1 protein level through USP22-CSN5-PD-L1 axis. In addition, USP22 depletion inhibited tumorigenesis and promoted T cell cytotoxicity. Besides, USP22 expression positively correlated with PD-L1 expression in human non-small cell lung cancer samples. Together, we identified a new regulator of PD-L1 and characterized the important role of USP22 in PD-L1 mediated immune evasion. Targeting USP22 might be a new solution to ICBT.

Video abstract

**Graphical abstract:**

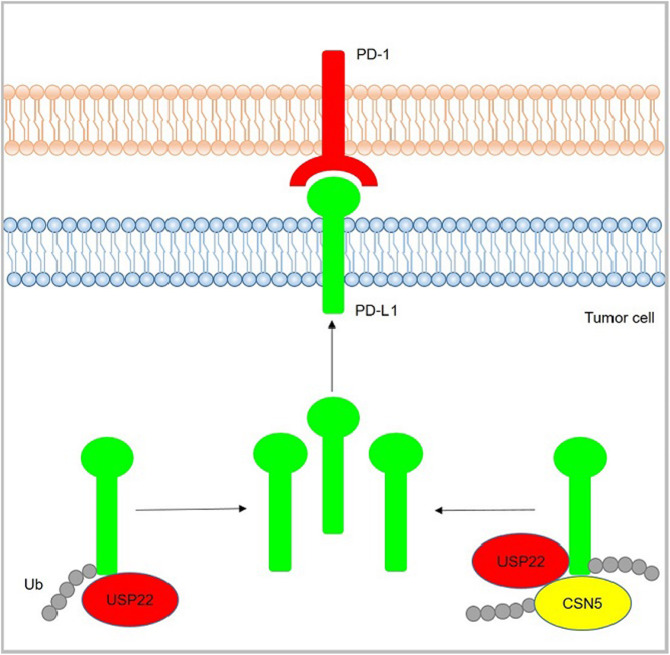

## Background

Today, tumor immunotherapy has convincingly been becoming a feasible approach to treat various cancers, e.g. blockade of checkpoint proteins in melanoma and non-small cell lung cancer (NSCLC), etc. [1]. PD-L1 (also known as CD274 or B7-H1) is a 33 kDa type I transmembrane glycoprotein that is involved in immune suppression. Many cancer cells evaded immune surveillance by overexpressing PD-L1 [2]. Besides, chemotherapeutic drugs could induce PD-L1 expression in various cancer types [3, 4]. PD-L1 can interact with its receptor PD-1 which is expressed on T cell surface, resulting in reduction of T cell proliferation and activation and thereafter cancer cell death mediated by T-lymphocyte [5]. Blocking these proteins with checkpoint inhibitors recovered recognition of cancer cells by T cells in the local immune system. The activated effector T cells eradicate cancer cells consequently [6].

However, the patient population that benefits from anti-PD-L1/PD-1 therapy is still limited to 20% in NSCLC, only a small proportion have long-term, durable responses [7–9]. Further understanding of the regulation of PD-L1 expression could be helpful for the improvement of anti-PD-L1/PD-1 therapy. Studies have shown that PD-L1 expression is regulated by signaling pathways such as PI3K, MAPK [10–13], transcriptional factors such as HIF1α, NF-κB, STAT3 [14–16] and epigenetic factors such as microRNAs [17]. Moreover, HIP1R targeted PD-L1 for lysosomal degradation [18]. CMTM6 appeared to regulate PD-L1 degradation through both proteasome and lysosome dependent way [19, 20]. Recent studies have shown that PD-L1 is also posttranslational regulated. For instance, palmitoylation stabilized PD-L1 by inhibiting ubiquitination and subsequent lysosomal degradation [21, 22]. GSK3β interacted with PD-L1 and induced phosphorylation-dependent proteasome degradation of PD-L1 by β-TrCP mediated ubiquitination [23]. CDK4 phosphorylated and stabilized SPOP, therefore, promoted cullin3-SPOP E3 ligase-induced PD-L1 ubiquitination during cell cycle [24]. In addition, CSN5 reduced PD-L1 ubiquitination and stabilized it [25, 26].

There are about 90 deubiquitinating enzymes (DUBs) in the human proteome consisting of five families: UCHs, USPs, OTUs, Josephins and JAMMs [27]. Ubiquitin-Specific Peptidase 22 (USP22) belongs to the subfamily, the ubiquitin-specific processing proteases (USPs). USP22 was regarded as an oncogene because it is overexpressed in malignant tumors of several tissues. Therefore, it can be used as a biomarker for predicting the recurrence and metastasis of malignance [28–30]. USP22 is a key subunit of the SAGA complex [31]. Besides histones, it could deubiquitinate TRF1, CCNB1, CCND1 and SIRT1 to regulate genes involved in metabolism, cell cycle and apoptosis [32–35]. USP22 stabilized these substrate proteins and inhibited their proteasome degradation. Of note, a very recent study revealed that USP22 deubiquitinated PD-L1 in HCC cells [36, 37].

COP9 signalosome 5 (CSN5) is the fifth component of the COP9 signalosome complex, which consists of eight subunits. CSN5 interacted with multiple molecules, such as c-Jun, p27, p53, Smad4, cullin1 [38–42]. CSN5 contained a conserved JAB1/MPN domain metalloenzyme (JAMM) motif, which possessed Need8 isopeptidase activity. Thus, CSN5 could regulate the activity of cullin-RING ligase (CRL) through deneddylation. Recently, CSN5 was reported to possess deubiquitination activity. Therefore, CSN5 participated in multiple signaling pathway and might possess multiple function during cancer progression.

In the present study, we demonstrated that USP22 regulated PD-L1 degradation in two ways. On the one hand, USP22 could directly regulate PD-L1 stability through deubiquitination. On the other hand, USP22 deubiquitinated CSN5 and regulated PD-L1 protein level through USP22-CSN5-PD-L1 axis. Therefore, our results demonstrated a new role of USP22 in regulating tumor immunosuppression.

## Methods

### Reagents and transfection

The primary antibodies for PD-L1 (PA5–28115; Thermo Fisher Scientific) and (MAB90781; R&D SYSTEMS), USP22 (SC-390585; Santa Cruz), CSN5 (SC-13157; Santa Cruz), HA (D110004; Sangon biotech) and (66006–1; proteintech), HIS (D291–3; MBL) T7 (PM022; MBL), MYC (C3956; sigma) and (M4439; sigma), FLAG (F1804; sigma) and (F7425; sigma), ACTB (A1978; sigma) were commercially available. MG132 (S2619) was purchased from selleck (shanghai, China). CHX (T1225) were purchased from TOPSCIENCE (shanghai, China). For detection of cell surface PD-L1, PE-conjugated mouse IgG1κ isotype control (12–4714-42) and PE-conjugated PD-L1 (12–5983-42) antibodies were purchased from eBioscience. For immunofluorescence experiment, Alexa Flour 488-anti-Mouse, Alexa Flour 555-anti-rabbit secondary antibodies were purchased from Invitrogen. For siRNA transfection, cells were seeded at 50% confluence and transfected with control or USP22 siRNA using Polyplus transfection reagent according to the manufacturer’s recommendations. For plasmid transfection, cells were seeded at 80% confluence and transfected with different plasmids using LipoMax transfection reagent according to the manufacturer’s recommendations. SiRNAs were synthesized by GenePharma (Shanghai, China). USP22 #1 and USP22 #4 siRNAs target the sequences 5′- CACAAAGCAGCTCACTATG-3′ and 5′- GCTGATCAACCTTGGGAAC − 3′, respectively. CSN5 #1 and CSN5 #2 siRNAs target the sequences 5′- GAGCUGUUGUGGAAUAAAU − 3′ and 5′- CCAGACUAUUCCACUUAA − 3′, respectively. Mus musculus Usp22 #1 and Usp22 #2 siRNAs target the sequences 5′-GAACAGACTTGAAGCATGT-3′ and 5′- GGGTCATTCATGAAGTTTA-3′, respectively. The pGiPZ plasmids used for stable cell line construction were a gift from Dr. Zhaoyuan Hou. All the plasmids used were cloned into pcDNA3.1 (+). For pcDNA3.1-PD-L1-K5R-T7 plasmid, K5R means 5 lysine amino acids in PD-L1 intracellular domain break into arginine. For pcDNA3.1-PD-L1-3NQ-FLAG plasmid, 3NQ means Substitution of each of the three asparagine (N) to glutamine (Q)— N192Q, N200Q, N219Q.

### Cell lines and cell culture

The HEK293FT cell line was cultured in DMEM medium 3.7 (Sigma Aldrich, 3.7 g NaHCO_3_/L) supplemented with 10% (v/v) fetal bovine serum (FBS) (Gibco). The B16-F10 cell line was cultured in DMEM medium 1.5 (Sigma Aldrich, 1.5 g NaHCO_3_/L) supplemented with 10% (v/v) fetal bovine serum (FBS) (Gibco). The A549, H157, H460, H1792, Calu-1, 95D and H1299 cell lines were cultured in RPMI 1640 (Sigma Aldrich) supplemented with 10% (v/v) FBS. All cell lines were originally obtained from the American Type Culture Collection (Manassas, VA) and were maintained at 37 °C in a humidified atmosphere consisting of 5% CO_2_ and 95% air.

### Western blot analysis

The preparation of whole-cell protein lysates and procedures used for the western blot analysis have been previously described [43]. The cells were harvested and rinsed with pre-chilled PBS. Then, the cells were lysed and centrifuged at 4 °C for 15 min. Samples of the whole-cell protein lysate were electrophoresed on a denaturing polyacrylamide slab gel and then transferred to a polyvinylidene fluoride (PVDF) membrane by electroblotting. The proteins were probed with the appropriate primary antibodies and subsequently the secondary antibodies. Antibody binding was detected by an HRP system according to the manufacturer’s protocol.

### Immunoprecipitation

The cells were lysed in precipitation lysis buffer (20 mM Tris-HCl, pH 7.5, 150 mM NaCl, 1 mM Na_2_EDTA, 1 mM EGTA, 1% Triton, 2.5 mM sodium pyrophosphate, 1 mM β-glycerophosphate, 1 mM Na_3_VO_4_) supplemented with protease inhibitors. Cell lysates were immunoprecipitated and analyzed by Western blot.

### Immunocytochemistry

For immunochemistry, cells were fixed and permeabilized in PHEMO buffer (0.025 M HEPES, 0.068 M PIPES, 0.003 M MgCl_2_·6H_2_O, 0.015 M EGTA·Na_2_, 10% DMSO, pH adjusted to 6.8. Additional reagents were added before use, with a final concentration as follows: 0.05% glutaraldehyde, 0.5% Triton X-100, 3.7% formaldehyde) at room temperature for 10 min, blocked in 3% BSA and then stained using primary antibodies. The secondary antibodies used were anti-rabbit Alexa Flour 555 dye conjugate and anti-mouse Alexa Flour 488 dye conjugate. Nuclei were stained with DAPI. After mounting, the cells were visualized using confocal microscope (ZEISS, LSM 700).

### Detection of cell surface PD-L1

Cells were seeded in six-well plates and allowed to reach 50% confluence. Then, cells were transfected with indicated siRNAs. Twenty-four hours post-transfection, 3 × 10^5^ cells were collected and washed with PBS containing 2% BSA. Cells were centrifuged and suspended in 70 μl PBS containing 2% BSA. 5 μl PE-conjugated PD-L1 antibody were added and incubated for 40 min on ice. Then, cells were washed and suspended in 200 μl PBS containing 2% BSA. Cells were analyzed by FACS. The whole process was protected from exposure to light.

### Animal experiments

1 × 10^6^ B16-F10 cells stably expressing CTRL or USP22 shRNA were suspended in 60 μl PBS and injected subcutaneously into female C57BL/6 mice (8 weeks). Mouse weight and tumor size were measured every second day. The tumors and spleens were extracted when the length of tumor reached 1 cm. Tumor volume was calculated using the formula: π/6 × length×width^2^.

### T cell-mediated tumor cell killing

Female C57BL/6 mice were injected with 1 × 10^6^ B16-F10 cells at 8 weeks, the spleens were obtained 12 days (tumor length reached 1 cm) after inoculation. The lymphocytes were extracted and stimulated with Concanavalin A (conA) (5 μg/ml) for 48 h. Then, lymphocytes were co-cultured with B16-F10 cells transfected with different siRNA under ConA (5 μg/ml) for 48 h. The supernatant were subjected to relative LDH release assay.

### Immunohistochemistry and scores

Specimens were deparaffinized and rehydrated, and antigen retrieval was performed in a microwave oven at 750 W for 30 min. To block endogenous peroxidase activity, the specimens were incubated in methanol containing 3% H_2_O_2_ at 37 °C for 30 min. Then, to avoid non-specific binding, the samples were incubated with preimmune serum at room temperature for 30 min. Next, 1:100 dilution of the primary antibody to PD-L1 (Rabbit monoclonal antibody, Catalogue Ab228462; Abcam, Cambridge, UK) and USP22 antibody at 1:50 dilution (Mouse monoclonal antibody, Catalogue sc390585; Santa Cruz, Dallas, Texas 75,220, U.S.A.) at 4 °C overnight. Following washes with phosphate-buffered saline (PBS) and incubation with a labeled polymer-HRP second antibody for 30 min, 3, 3-diaminobenzidine tetrachloride (DAB) was applied to initiate the colorimetric reaction. All sections were then counterstained in hematoxylin. Immunohistochemical staining of sections was scored at 200× magnification light microscopy. USP22 positive expression in the cytoplasm and nuclear of cancer cells. The staining evaluation for USP22 was based on a semi-quantitative method described as follows [44]: Staining intensity for USP22 was scored as 0 (negative), 1 (weak), 2 (moderate), and 3 (strong). Staining extent was scored as 0 (0%), 1 (1–25%), 2(26–50%), 3(51–75%), and 4 (76–100%), depending on the percentage of positive-stained cells. The sum of the staining intensity and the staining extent scores were ranged from 0 to 7, with negative staining (0–1) and positive expression (2–7). While for PD-L1 staining, cell surface membrane staining > 5% was considered positive. Agreement between the two evaluators was 95%, and all scoring discrepancies were resolved through discussion.

### Statistical analysis

GraphPad Prism software was used for statistical analysis. All data were presented as the mean ± SD. Differences between groups were identified using two-sided Student’s t-test. One way ANOVA were carried out for animal experiments. USP22 and PD-L1 relation in human non-small cell lung cancer samples were analyzed using Chi-square test. *P* < 0.05 was considered statistically significant.

## Results

### USP22 regulated PD-L1 protein level in NSCLC cells

**Fig. 1:**
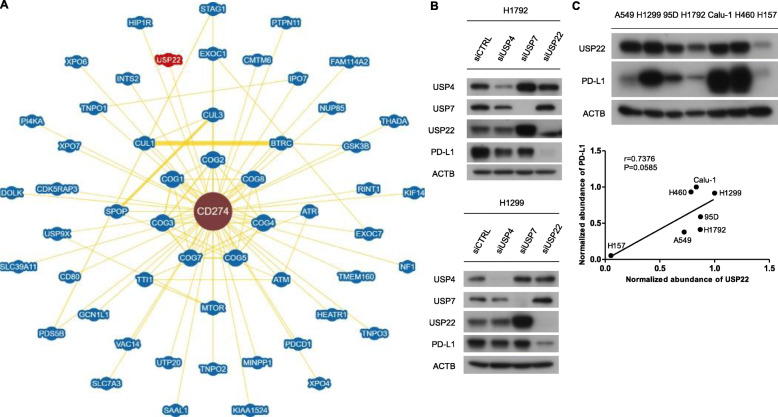
USP22 is a potential partner and regulator of PD-L1. **a** Interaction network of PD-L1 analyzed in BioGrid database. **b** H1792, H1299 cells were transfected with control (CTRL) or USP4, USP7, USP22 siRNA for 24 h and then subjected to western blot analysis. **c** Western blot analysis of USP22 and PD-L1 expression in seven NSCLC cell lines. Relative intensity of USP22 and PD-L1 were normalized to ACTB. The pearson correlation coefficient for the protein expression were presented

In order to discover new regulators of PD-L1, we searched potential interactors of PD-L1 in BioGRID database. We found that USP22 is a candidate regulator according to mass spectrometry data (Fig. 1a). To convince the specificity of USP22, we knocked down USP4, USP7 and USP22 in H1792 and H1299 cells. The results showed that depletion of USP22 but not USP4 or USP7 reduced PD-L1 protein level (Fig. 1b), which suggested that USP22 is a specific regulator of PD-L1. After screening USP22 and PD-L1 protein expression in seven NSCLC cell lines, we found that PD-L1 protein level positively correlated with USP22 expression (Fig. 1c). These data implied that USP22 might be a potent regulator of PD-L1.

**Fig. 2:**
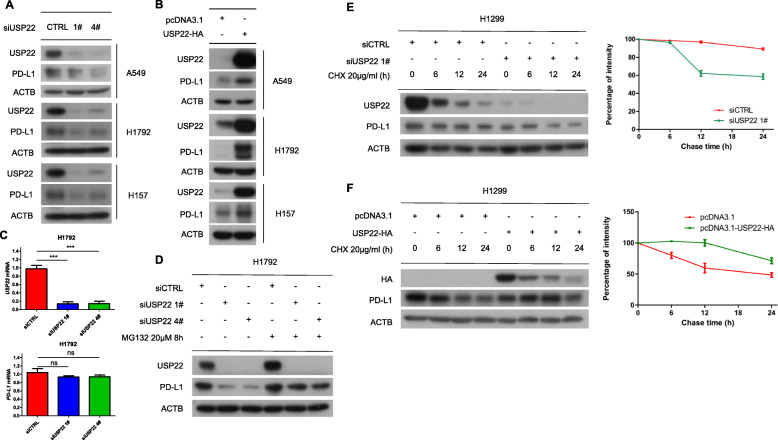
USP22 influences PD-L1 protein stabilization. **a** A549, H1792, H1299 cells were transfected with control (CTRL) or USP22 siRNA for 24 h and then subjected to western blot analysis. **b** A549, H1792, H1299 cells were transfected with pcDNA3.1-USP22-HA plasmid for 24 h and then subjected to western blot analysis. pcDNA3.1 empty vector was transfected as control. **c** H1792 cells were transfected with control (CTRL) or USP22 siRNA for 24 h before RNA was extracted and subjected to reverse transcript PCR (RT-PCR). After agarose gel electrophoresis, mRNA levels were determined by image J. Error bars represent SD (*n* = 3), ***P < 0.001. **d** PD-L1 protein level was measured upon USP22 knockdown with or without proteasome inhibitor MG132 in H1792 cells. **e** H1299 cells were transfected with control (CTRL) or USP22 siRNA for 24 h followed by cycloheximide (CHX) (20 μg/ml) for indicated time. Relative protein abundance was measured by image J. Experiments were repeated three times. **f** H1299 cells were transfected with pcDNA3.1 empty vector or pcDNA3.1-USP22-HA plasmid for 24 h followed by cycloheximide (CHX) (20 μg/ml) for indicated time. Relative protein abundance was measured by image J. Experiments were repeated three times

To verify our hypothesis, we interfered USP22 expression with distinct siRNAs in three NSCLC cell lines such as A549, H1792 and H157. Depletion of USP22 caused downregulation of both endogenous and exogenous PD-L1 protein level (Fig. 2a, Additional file 1: Fig. S1A). Similar results were observed in breast and colorectal cancer cell lines (Additional file 1: Fig. S1B). Consistently, ectopic expression of USP22 elevated PD-L1 protein level (Fig. 2b, Additional file 1: Fig. S1C). Based on these results, we considered USP22 as a regulator of PD-L1. Now that USP22 is a subunit of SAGA transcriptional coactivator complex, we therefore determined if USP22 regulated PD-L1 at transcriptional level. However, PD-L1 mRNA was not affected upon USP22 depletion (Fig. 2c, Additional file 1: Fig. S1D). Therefore, we asked whether USP22 regulated PD-L1 protein degradation. Proteasome inhibitor MG132 rescued PD-L1 downregulation induced by USP22 depletion (Fig. 2d, Additional file 1: Fig. S1E), indicating that USP22 depletion induced PD-L1 proteasome degradation. Furthermore, we used Cycloheximide (CHX), an inhibitor of protein synthesis, to explore whether USP22 affected PD-L1 stability. Our data showed that USP22 depletion decreased the stability of PD-L1 while USP22 overexpression prolonged PD-L1 protein half-life (Fig. 2e, Fig. 2f). Taken together, our results indicated that USP22 participated in proteasome degradation of PD-L1 in human cancer cells.

### USP22 physically interacted with PD-L1

**Fig. 3:**
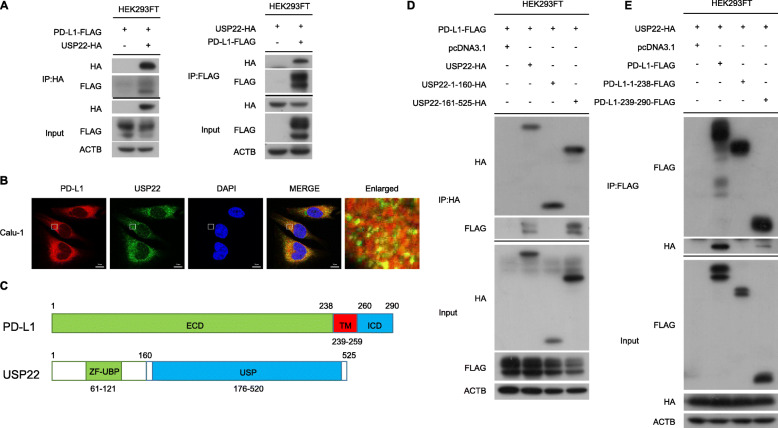
USP22 physically interacts with PD-L1. **a** Co-immunoprecipitation were carried out using either anti-HA or anti-FLAG antibody in HEK293FT cells. **b** Immunofluorescence staining of endogenous PD-L1 and USP22. Scale bar, 10 μm. **c** Schematic diagram of PD-L1 and USP22. ECD, extracellular domain; ICD, intracellular domain; TM, transmembrane domain. **d** The interaction of PD-L1 with fragments of USP22. **e** The interaction of USP22 with fragments of PD-L1

Now that USP22 could regulate PD-L1 protein level, we asked whether USP22 interacted with PD-L1. We performed co-immunoprecipitation assays in HEK293FT and H157 cells respectively, and we found that USP22 interacted with PD-L1 (Fig. 3a, Additional file 1: Fig. S2A). Furthermore, immunostaining experiment confirmed the results in Calu-1 cells (Fig. 3b). Thus, we validated the interaction between USP22 and PD-L1. USP22 is composed of two domains, the zinc finger at the N-terminal and the catalytic domain at the C-terminal [45]. PD-L1 consisted of ECD (extracellular domain), TM (transmembrane domain) and ICD (intracellular domain) (Fig. 3c). Further analysis revealed that the catalytic C-terminal fragment of USP22 interacted with PD-L1 (Fig. 3d). Besides, PD-L1 ICD domain possess the binding ability with USP22 (Fig. 3e). In general, our results suggested that the regulatory role of USP22 on PD-L1 might depend on their physical interaction.

### USP22 modulated deubiquitination of PD-L1 in cancer cells

**Fig. 4:**
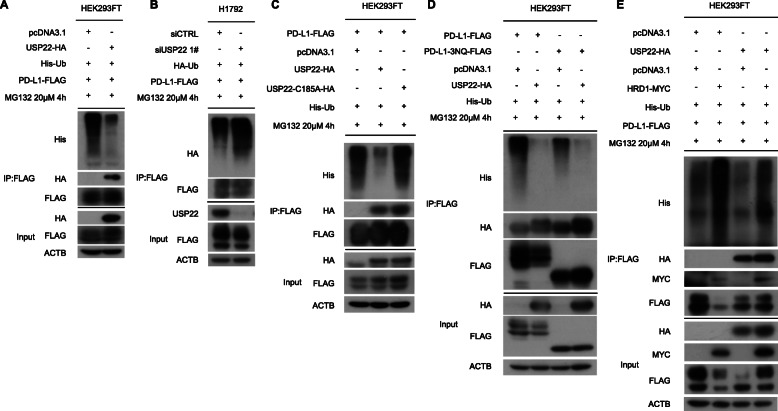
USP22 induces PD-L1 deubiquitination. **a** HEK293FT cells were transfected with indicated constructs. The impact of USP22 overexpression on PD-L1 ubiquitination were tested. **b** PD-L1 ubiquitination were conducted upon USP22 depletion. **c** Wild type or loss of function mutation (C185A) of USP22 were transfected to measure PD-L1 ubiquitination. **e** The effects of USP22 and HRD1 on PD-L1 ubiquitination. **d** USP22 was introduced to analyze glycosylated and non-glycosylated PD-L1 ubiquitination in HEK293FT cells. PD-L1-3NQ-FLAG means Substitution of each of the three asparagine (N) to glutamine (Q)— N192Q, N200Q, N219Q, which is critical for PD-L1 glycosylation

Given that USP22 is a deubiquitinating enzyme, we wondered if USP22 regulated PD-L1 deubiquitination. To this end, we performed co-IP experiment in HEK293FT and H1299 cells. As expected, USP22 significantly reduced PD-L1 poly-ubiquitination in cancer cells (Fig. 4a, Additional file 1: Fig. S2B). Consistently, PD-L1 poly-ubiquitination was elevated after USP22 depletion (Fig. 4b). Notably, active site dead mutant USP22-C185A failed to suppress PD-L1 poly-ubiquitination without affecting its interaction with PD-L1 (Fig. 4c). While K48-linked ubiquitin chain is mainly related to proteasome degradation, our result demonstrated that USP22 removed K6, K11, K27, K29, K33, K63-linked ubiquitin chain of PD-L1 (Additional file 1: Fig. S2C). Now that glycosylation of PD-L1 is essential for immune suppression [46], we asked whether PD-L1 glycosylation affected USP22-induced PD-L1 deubiquitination. However, USP22 suppressed ubiquitination of both glycosylated and non-glycosylated PD-L1 (Fig. 4d). To explore which domain of PD-L1 is responsible for USP22-mediated deubiquitination, we replaced all five lysine residues with arginines (PD-L1-K5R) in PD-L1 intercellular domain. USP22 knockdown failed to decrease mutant PD-L1 protein level, which indicated that the ubiquitination site resided in PD-L1 intercellular domain (Additional file 1: Fig. S3A). HRD1 (also known as SYVN1) was an E3 ubiquitin ligase that participated in ubiquitination and degradation of PD-L1 under endoplasmic-reticulum-associated protein degradation (ERAD) [47]. We wondered if the relationship between USP22 and HRD determined PD-L1 ubiquitination level and degradation. Actually, USP22 reduced HRD1-induced PD-L1 ubiquitination and protected PD-L1 from HRD1-mediated degradation (Fig. 4e, Additional file 1: Fig. S3B). Together, our results suggested that USP22 regulated PD-L1 protein stabilization through its deubiquitination activity.

### USP22 interacted with CSN5 and deubiquitinated CSN5

**Fig. 5:**
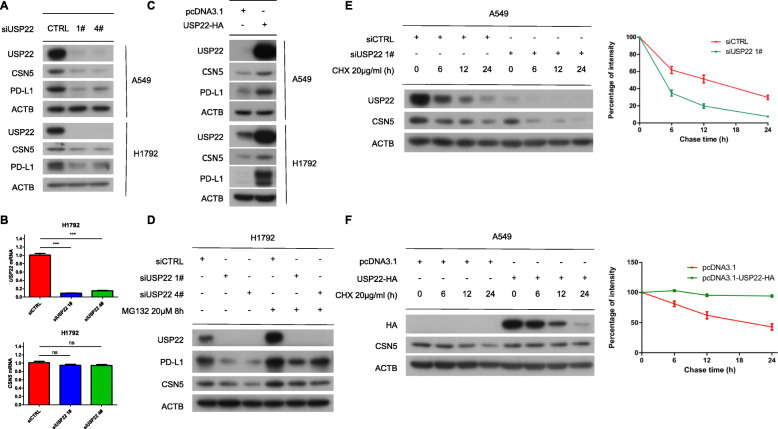
USP22 enhances the stability of CSN5. **a** A549, H1792 cells were transfected with control (CTRL) or USP22 siRNA for 24 h and then subjected to western blot analysis. **b** H1792 cells were transfected with control (CTRL) or USP22 siRNA for 24 h before RNA was extracted and subjected to reverse transcript PCR (RT-PCR). Protein and mRNA level of USP22, CSN5, PD-L1 were assesses using western blot and agarose gel electrophoresis respectively. mRNA levels were determined by image J. Error bars represent SD (*n* = 3), ***P < 0.001. Statistical differences were determined by two-sided Student’s t-test. **c** A549, H1792 cells were transfected with pcDNA3.1-USP22-HA plasmid for 24 h and then subjected to western blot analysis. pcDNA3.1 empty vector was transfected as control. **d** CSN5 protein level were measured upon USP22 knockdown with or without proteasome inhibitor MG132 in H1792 cells. **e** A549 cells were transfected with control (CTRL) or USP22 siRNA for 24 h followed by cycloheximide (CHX) (20 μg/ml) for indicated time. Relative protein abundance was measured by image J. Experiments were repeated three times. **f** A549 cells were transfected with pcDNA3.1 empty vector or pcDNA3.1-USP22-HA plasmid for 24 h followed by cycloheximide (CHX) (20 μg/ml) for indicated time. Relative protein abundance was measured by image J. Experiments were repeated three times

CSN5 was reported to be a deubiquitinating enzyme for PD-L1 [25], which arose our interest to focus on the interplay between USP22 and CSN5. To this end, we found that USP22 knockdown decreased CSN5 protein level without affecting CSN5 mRNA level (Fig. 5a, Fig. 5b, Additional file 1: Fig. S4A). Consistently, ectopic expression of USP22 upregulated CSN5 protein level (Fig. 5c). However, interference with CSN5 expression had no effect on USP22 protein level (Additional file 1: Fig. S4B). Similarly, USP22 depletion induced proteasome degradation of CSN5 (Fig. 5d). While using cycloheximide (CHX) to inhibit protein synthesis, USP22 depletion indeed reduced CSN5 protein half-life while USP22 overexpression enhanced CSN5 stability (Fig. 5e, Fig. 5f). These data implied that USP22 stabilized CSN5 while CSN5 could not affect USP22 protein level.

**Fig. 6:**
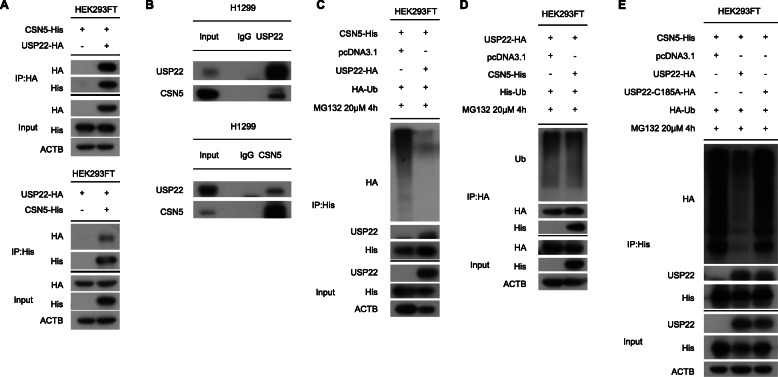
USP22 targets CSN5 via deubiquitination. **a** HEK293FT cell lysates subjected to IP under non-denaturing conditions using either anti-HA or anti-His antibody. **b** IP assays of H1299 cells using anti-USP22 or anti-CSN5 antibody. **c** HEK293FT cells were transfected with indicated constructs. The impact of USP22 overexpression on CSN5 ubiquitination were tested. **d** The impact of CSN5 overexpression on USP22 ubiquitination was tested in HEK293FT cells. **e** In HEK293FT cells, wild type or loss of function mutation (C185A) of USP22 were transfected to measure CSN5 ubiquitination

Since CSN5 bound to PD-L1 [25], we wondered if USP22 interacted with CSN5 physically. As expected, USP22 interacted with CSN5 exogenously and endogenously (Fig. 6a, Fig. 6b). Immunostaining experiment revealed co-localization of USP22 and CSN5 (Additional file 1: Fig. S4C). Besides, USP22 could remove poly-ubiquitin chain of CSN5, while CSN5 seemed no effect on USP22 ubiquitination (Fig. 6c, Fig. 6d). In contrast to wild type USP22, catalytically inactive mutant USP22-C185A failed to reduce CSN5 ubiquitination (Fig. 6e). To extend our findings, we performed a thorough deubiquitination assay of CSN5 by USP22 with a series of ubiquitin mutants. Similar to PD-L1, USP22 removed K6, K11, K27, K29, K33, K63-linked ubiquitin chain of CSN5 (Additional file 1: Fig. S4D). Taken together, USP22 interacted with CSN5 and stabilized CSN5 protein through its deubiquitination activity.

### USP22 coordinated with CSN5 to regulate PD-L1

**Fig. 7:**
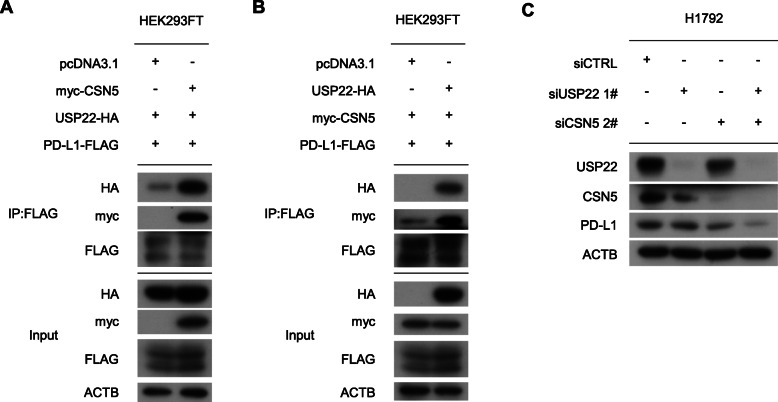
USP22 collaborates with CSN5 to regulate PD-L1 (**a**, **b**) USP22, CSN5, PD-L1 plasmids were transfected as indicated. **c** H1792 cells were transfected with USP22 or/and CSN5 siRNA. USP22, CSN5, PD-L1 protein level were assessed by western blot

Based on our findings that USP22 deubiquitinated CSN5 [25] and the previous findings that CSN5 deubiquitinated PD-L1, it’s reasonable to hypothesize that USP22 might modulated PD-L1 through the USP22-CSN5-PD-L1 axis. Thus, we thought USP22 regulated PD-L1 ubiquitination in two ways: first, USP22 directly removed PD-L1 ubiquitination; second, USP22 modulated CSN5 and regulated PD-L1 ubiquitination through USP22-CSN5-PD-L1 axis. Since both USP22 and CSN5 could bind to PD-L1 and affected its ubiquitination [25], we are wondering what the relationship there was between USP22 and CSN5 to influence PD-L1 stability. Our results showed that CSN5 enhanced the interaction between USP22 and PD-L1 (Fig. 7a). Furthermore, USP22 also facilitated the interaction between CSN5 and PD-L1 (Fig. 7b). These data showed that USP22 and CSN5 enhanced the interaction of PD-L1 with the other. Then, we simultaneously knocked down USP22 and CSN5 in H1792 cells. We found that simultaneous USP22 and CSN5 knockdown further downregulated PD-L1 protein level compared with single knockdown (Fig. 7c). Collectively, these results indicated that USP22 coordinated with CSN5 to regulate PD-L1.

### Inhibition of Usp22 suppressed tumorigenesis

**Fig. 8:**
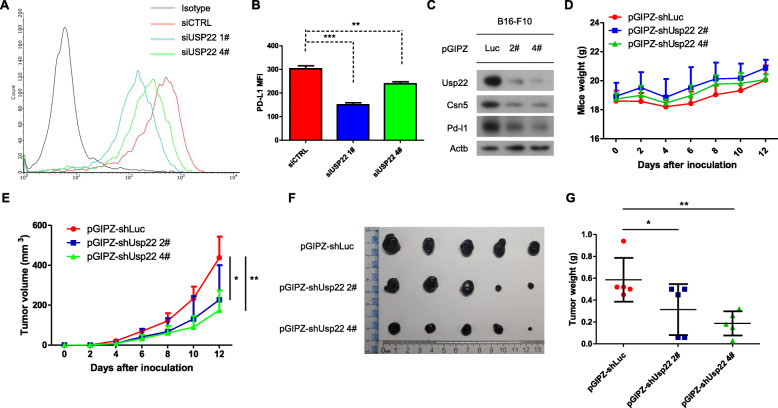
Inhibition of Usp22 suppresses tumorigenesis. **a** Cell surface PD-L1 level was measured after treatment with CTRL or USP22 siRNA in Calu-1 cell line. **b** Mean fluorescence intensity (MFI) was analyzed. Values are means ±SD, *n* = 3. Statistical differences were determined by two-sided Student’s t-test, **P < 0.01. **c** USP22, CSN5, PD-L1 protein level were assessed in pGIPZ-shluc and pGIPZ-shUSP22 infected B16-F10 cells. **d** B16-F10 tumor grow over time after transduction with the indicated lentiviruses. Tumor weight were measured every second day. Error bars represent SD (*n* = 5). **e** Tumor volume were assessed in (**b**). Error bars represent SD (*n* = 5). **P < 0.01, *P < 0.05, Statistical differences were determined by one-way ANOVA. **f** Representative images of the tumors in (c) taken 12 days after inoculation. **g** Tumor weight were measured. Error bars represent SD (*n* = 5). **P < 0.01, *P < 0.05, Statistical differences were determined by one-way ANOVA

To validate the role of USP22 in PD-L1 regulation, we therefore determined cell surface PD-L1 expression. Depletion of USP22 reduced membrane PD-L1 level, as revealed by flow cytometry (Fig. 8a, Fig. 8b). PD-L1 function as immune checkpoint to inhibit cytotoxic T cell-mediated tumor killing, we asked whether USP22 depletion suppressed tumorigenesis. On the basis of current clinical data of PD-1/PD-L1 pathway blockade, bladder cancer, melanoma, mismatch repair–deficient colorectal cancer, and certain hematopoietic malignancies may be among the most responsive cancer types [48]. Besides, B16-F10 exhibited higher PD-L1 level (mouse melanoma cancer cell line) compared to LLC1 (mouse lung cancer cell line) and MC38 (mouse colorectal cancer cell line) (data not shown). Therefore, we chose B16-F10 to validate the role of USP22 in PD-L1 mediated immune checkpoint blockade. Stable USP22 knockdown B16-F10 cells showed decreased CSN5 and PD-L1 expression (Fig. 8c). USP22 depletion suppressed tumorigenesis without altering mouse weight (Fig. 8d). Meanwhile, tumor volume and tumor weight is significantly reduced upon stable USP22 knockdown (Fig. 8e-g). These results supported the notion that USP22 might regulate PD-L1 to suppress tumorigenesis.

### USP22 enhanced immunosuppression via PD-L1

**Fig. 9:**
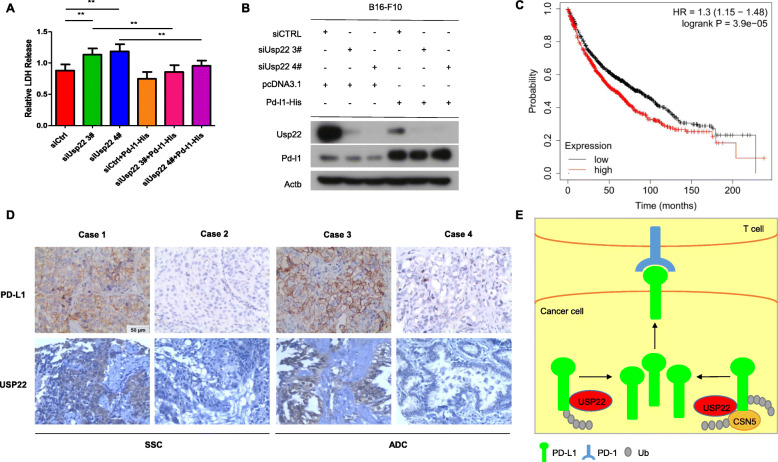
Effects of Usp22 on T cell cytotoxicity. **a** T cell killing assay of B16-F10 transfected with Pd-l1-His plasmids and Usp22 siRNA. Lymphocytes were isolated from C57BL/6 mice. Lymphocytes were stimulated with 5 μg/ml Concanavalin A (conA) for 48 h before co-cultured with B16-F10 cells for another 48 h in the presence of 5 μg/ml conA. The supernatant was used for LDH release assay. Error bars represent SD (*n* = 4). **P < 0.01, *P < 0.05, Statistical differences were determined by two sided Student’s t-test. **b** Western blot analysis of B16-F10 cells illustrated in (**a**). **c** KM plotter analysis of the relation between USP22 gene expression and prognosis (OS) in lung cancer patients. **d** Representative immunohistochemical staining results for US22 and PD-L1 in human lung adenocarcinoma (ADC) and squamous cell carcinoma (SCC). Scale bar, 50 μm (**e**) Proposed modal of USP22-mediated PD-L1 regulation. On the one hand, USP22 directly removed PD-L1 ubiquitination; on the other hand, USP22 modulated CSN5 through deubiquitination and regulated PD-L1 ubiquitination through USP22-CSN5-PD-L1 axis

Immune checkpoint blockade therapies (ICBTs) targeting PD-L1 and PD-1 have exhibited prominent clinical benefits in multiple tumor types. We therefore asked whether USP22 influenced immunosuppression caused by PD-L1-PD1 axis. To perform T cell-mediated cell killing assay, lymphocytes were separated from tumor-bearing mice. T cells were stimulated with Concanavalin A (conA) before co-cultured with B16-F10 cells transfected with indicated constructs. We found that USP22 knockdown enhanced LDH release, indicating that T cell-mediated cell killing in B16-F10 cells were elevated, while simultaneous ectopic expression of PD-L1 somehow rescued the effect (Fig. 9a, Fig. 9b). Low USP22 gene expression is associated with better prognosis in lung cancer patients according to KM poltter database (Fig. 9c). The regulation of USP22 on PD-L1 might partially explain the correlation. To further validate the pathologic relevance of USP22 and PD-L1, we studied the expression of USP22 and PD-L1 in 241 human non-small cell lung cancer samples using immunohistochemical staining. USP22 was detected in 40 (59.7%) of the 67 specimens with high PD-L1 expression but in only 66 (37.9%) of the 174 specimens with low PD-L1 expression, indicating that there is a positive correlation between USP22 and PD-L1 expression (Fig. 9d, Additional file 2: Table S1). These data suggested that USP22 might be a target to improve the efficiency of cancer treatments based on ICBT.

## Discussion

PD-L1 is expressed in cancer cells and antigen presenting cells, and plays an important role in ICBT [2, 49]. Recently, PD-L1 was found in tumor-derived exosomes, which inhibited anti-PD-L1/PD-1 therapy [43, 50]. Several works have focused on post-transcription modifications of PD-L1. Here, we found that USP22 regulated PD-L1 protein level. Furthermore, we confirmed the interaction between USP22 and PD-L1 both in HEK293FT and NSCLC cells. Immunofluorescence results also supported the findings. Unlike transcription factors that regulated PD-L1 at the transcriptional level, USP22 knockdown mediated PD-L1 degradation at the post-translational level through ubiquitin-proteasome pathway. Our results demonstrated that USP22 removed poly-ubiquitin chains of PD-L1 in a kinase dependent way. Based on their conjugation style, eight types of ubiquitin linkage had been identified: K6, K11, K27, K29, K33, K48, K63, and linear ubiquitination [51, 52]. USP22 was reported to modulate K63-linked rather than K48-linked ubiquitination of FBP1 [53]. Consistently, USP22 removed K6, K11, K27, K29, K33, K63-linked poly-ubiquitin chains of PD-L1. N-linked glycosylation of PD-L1 is important for PD-L1 mediated immunosuppression. On the one hand, PD-L1 is stabilized by glycosylation [23, 54, 55]. On the other hand, glycosylation of PD-L1 is necessary for PD-L1/PD-1 interaction [46]. However, USP22 deubiquitinated both glycosylated and non-glycosylated PD-L1, indicating that glycosylation seemed not involved in USP22 mediated PD-L1 regulation, and USP22 might be a powerful target for PD-L1/PD-1 blockade therapy. Substitution of lysines with arginines in PD-L1 intercellular domain prevented USP22 depletion-induced downregulation of PD-L1, which suggested that USP22 stabilized PD-L1 through deubiquitination of its intercellular lysine amino acid. Considering that USP22 is a stem cell marker [31], combination treatment with PD-L1/PD-1 blockade antibody and USP22 inhibitor might eliminate cancer stem cell.

We found that USP22 regulated CSN5 stability through its deubiquitination activity. However, CSN5 couldn’t affect USP22 protein turnover. Of note, USP22 also removed K6, K11, K27, K29, K33, K63-linked ubiquitin chain of CSN5, which implied that USP22 might possess conserved substrate activity pattern. Previous work reported that CSN5 could regulate PD-L1 stability through its deubiquitination activity [25]. Our results showed that USP22 was also a deubiquitinase for PD-L1. Moreover, USP22 could direct deubiquitinated CSN5. Together, we found that USP22 regulated PD-L1 in two ways. First, USP22 directly deubiquitinated and regulated PD-L1. Second, USP22 deubiquitinated CSN5 and regulated PD-L1 protein level through USP22-CSN5-PD-L1 axis (Fig. 9d). We have also considered the possibility that USP22 regulated PD-L1 expression via CSN5. In the Fig. 7c, USP22 and CSN5 double knock-down further reduced PD-L1 protein level compared to CSN5 single knockdown. This data showed that USP22 could regulate PD-L1 in the absence of CSN5, which supported our model. In addition, USP22 could facilitated the interaction between CSN5 and PD-L1, and CSN5 promoted the interaction between USP22 and PD-L1. Considering that USP22 could enhance CSN5 protein level, there might exist a positive feedback mechanism. That is, USP22 enhanced CSN5 stability and in turn facilitated the interaction of USP22 and PD-L1, indicating that USP22 and CSN5 worked cooperatively to regulate PD-L1.

Now that CSN5 interacted with various proteins and participated in multiple signaling pathway, USP22 might regulate different cell activity via CSN5. However, how USP22 sense tumor microenvironment signals to regulate PD-L1 remains unknown. Together, we provided molecular insights into the mechanisms that control the homeostasis of PD-L1.

Functionally, USP22 depletion suppressed tumorigenesis in B16-F10 cell line. Furthermore, USP22 depletion down-regulated PD-L1 protein level while promoted T cell-mediated cell killing. The positive correlation of USP22 and PD-L1 expression in human tumor samples implied the possibility that USP22 targeted therapy might be in favor of cancer treatment via PD-L1 regulation. USP22 inhibitor might improve the efficacy of PD-L1/PD-1 antibodies by suppressing PD-L1 protein level. Combination of PD-L1/PD-1 blockade therapy with targeted therapy or chemotherapy have been proved to improve outcomes rather than monotherapy [56, 57]. Thus, combination of USP22 targeted therapy with other targeted therapy is a promising solution. Taken together, our study characterized the role of USP22 in immunosuppression via regulation of PD-L1, and USP22 might be a target for ICBT.

## Conclusions

In summary, our work identified USP22 as a new regulator of PD-L1. USP22 reduced PD-L1 ubiquitination and protected PD-L1 from proteasome-mediated degradation. Besides, USP22 interacted and stabilized CSN5 through deubiquitination. On the one hand, USP22 could regulate PD-L1 protein level through direct deubiquitination. On the other hand, USP22 modulated PD-L1 through the USP22-CSN5-PD-L1 axis. Both USP22 and CSN5 could facilitate the interaction of PD-L1 with the other one, which suggested that USP22 and CSN5 worked cooperatively to regulate PD-L1. Moreover, USP22 depletion suppressed tumorigenesis and promoted T cell-mediated cell killing. Besides, USP22 expression positively correlated with PD-L1 expression in human non-small cell lung cancer samples. This regulation is vital for evade immune surveillance via PD-L1/PD-1 checkpoint blockade.

## Supplementary information

**Additional file 1: Figure S1.** USP22 influences PD-L1 protein abundance. **Figure S2.** USP22 deubiquitinates PD-L1. **Figure S3.** USP22 deubiquitinates PD-L1. **Figure S4.** USP22 targets CSN5 for Deubiquitination.

**Additional file 2: Table S1.** Correlations between expression levels of USP22 and PD-L1

## Data Availability

The datasets used/analyzed to support the conclusion of article are available from the corresponding author upon reasonable request.

## Abbreviations

PD-L1: Programmed Cell Death 1 Ligand 1
USP22: Ubiquitin Specific Peptidase 22
CSN5: COP9 Signalosome Subunit 5
CHX: Chlorhexidine
NSCLC: Non-small cell lung cancer
ICBT: Immune checkpoint blockade therapy
