# Supplementary material for: The deubiquitinase USP22 regulates PD-L1 degradation in human cancer cells
Source: Cell Commun Signal. 2020 Jul 14;18:112. doi: 10.1186/s12964-020-00612-y (PMC7362500; doi:10.1186/s12964-020-00612-y)
Supplement: Supplementary file 2 — Additional file 1: Figure S1. USP22 influences PD-L1 protein abundance. Figure S2. USP22 deubiquitinates PD-L1. Figure S3. USP22 deubiquitinates PD-L1. Figure S4. USP22 targets CSN5 for Deubiquitination. [file 12964_2020_612_MOESM2_ESM.zip › Fig. S1-4.pptx]

## Slide 1
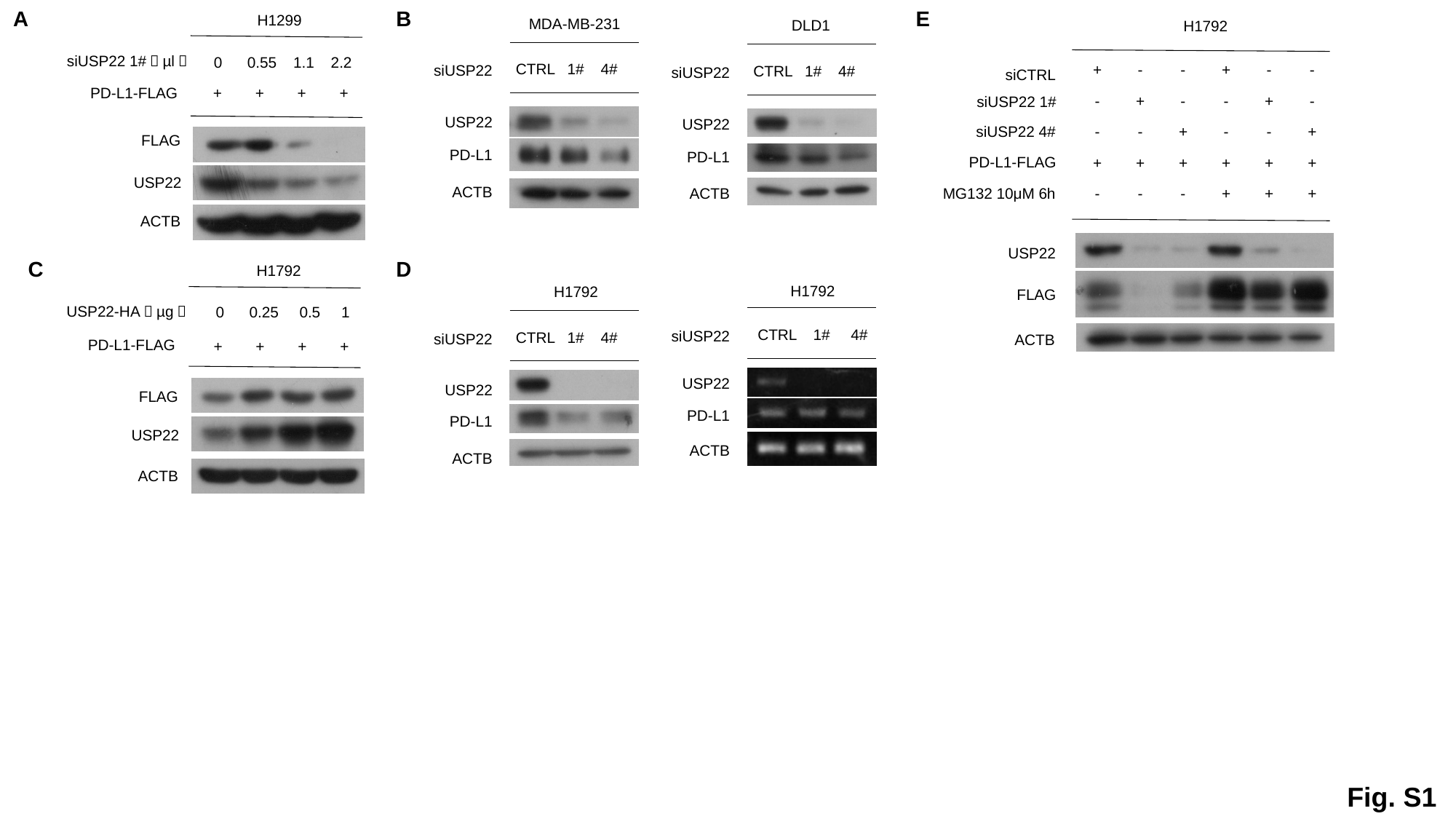

A
B
E
 H1299
 MDA-MB-231
 DLD1
 H1792
siUSP22 1#（µl）
 0 0.55 1.1 2.2
 CTRL 1# 4#
siUSP22
| + | - | - | + | - | - |
| --- | --- | --- | --- | --- | --- |
| - | + | - | - | + | - |
| - | - | + | - | - | + |
| + | + | + | + | + | + |
| - | - | - | + | + | + |
 CTRL 1# 4#
siUSP22
 siCTRL
PD-L1-FLAG
 + + + +
 siUSP22 1#
USP22
USP22
 siUSP22 4#
 FLAG
 PD-L1
 PD-L1
PD-L1-FLAG
 USP22
 ACTB
 ACTB
 MG132 10μM 6h
 ACTB
 USP22
C
D
 H1792
 H1792
 H1792
FLAG
USP22-HA（µg）
 0 0.25 0.5 1
 CTRL 1# 4#
siUSP22
 CTRL 1# 4#
siUSP22
ACTB
PD-L1-FLAG
 + + + +
USP22
USP22
 FLAG
 PD-L1
 PD-L1
 USP22
 ACTB
 ACTB
 ACTB
Fig. S1

## Slide 2
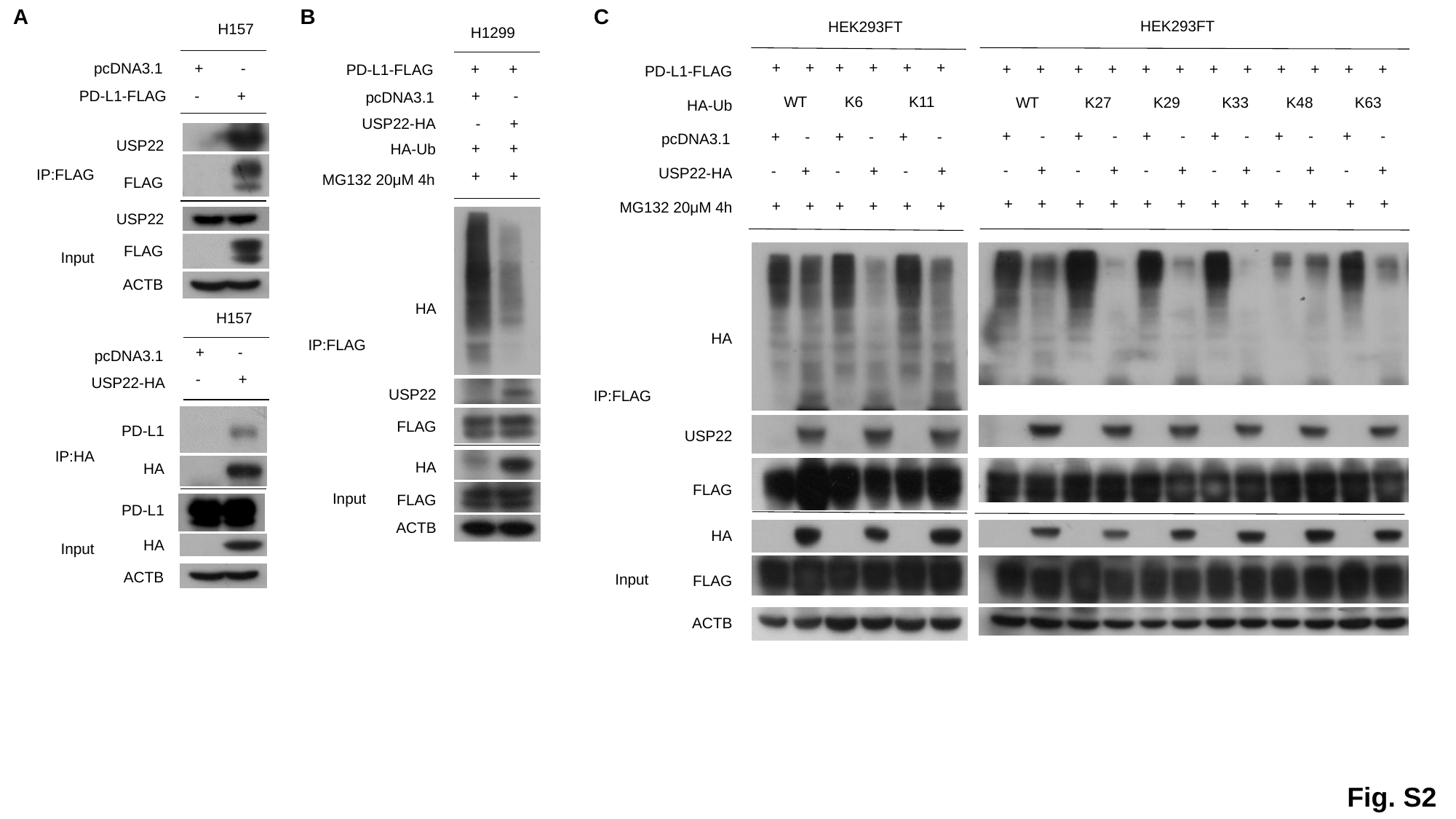

A
B
C
 HEK293FT
 HEK293FT
 H157
 H1299
 + + + + + +
pcDNA3.1
 + -
+ + + + + + + + + + + +
 + +
PD-L1-FLAG
PD-L1-FLAG
 - +
PD-L1-FLAG
 + -
pcDNA3.1
 WT K6 K11
 WT K27 K29 K33 K48 K63
HA-Ub
USP22-HA
 - +
 + - + - + - + - + - + -
 + - + - + -
pcDNA3.1
 USP22
HA-Ub
 + +
- + - + - + - + - + - +
 - + - + - +
USP22-HA
IP:FLAG
 + +
 MG132 20μM 4h
 FLAG
+ + + + + + + + + + + +
 + + + + + +
MG132 20μM 4h
 USP22
 FLAG
 Input
 ACTB
 HA
 H157
 HA
 IP:FLAG
 + -
pcDNA3.1
 - +
USP22-HA
 USP22
 IP:FLAG
 FLAG
 PD-L1
USP22
 IP:HA
 HA
HA
FLAG
 Input
 FLAG
 PD-L1
 ACTB
 HA
 HA
 Input
 ACTB
Input
 FLAG
 ACTB
Fig. S2

## Slide 3
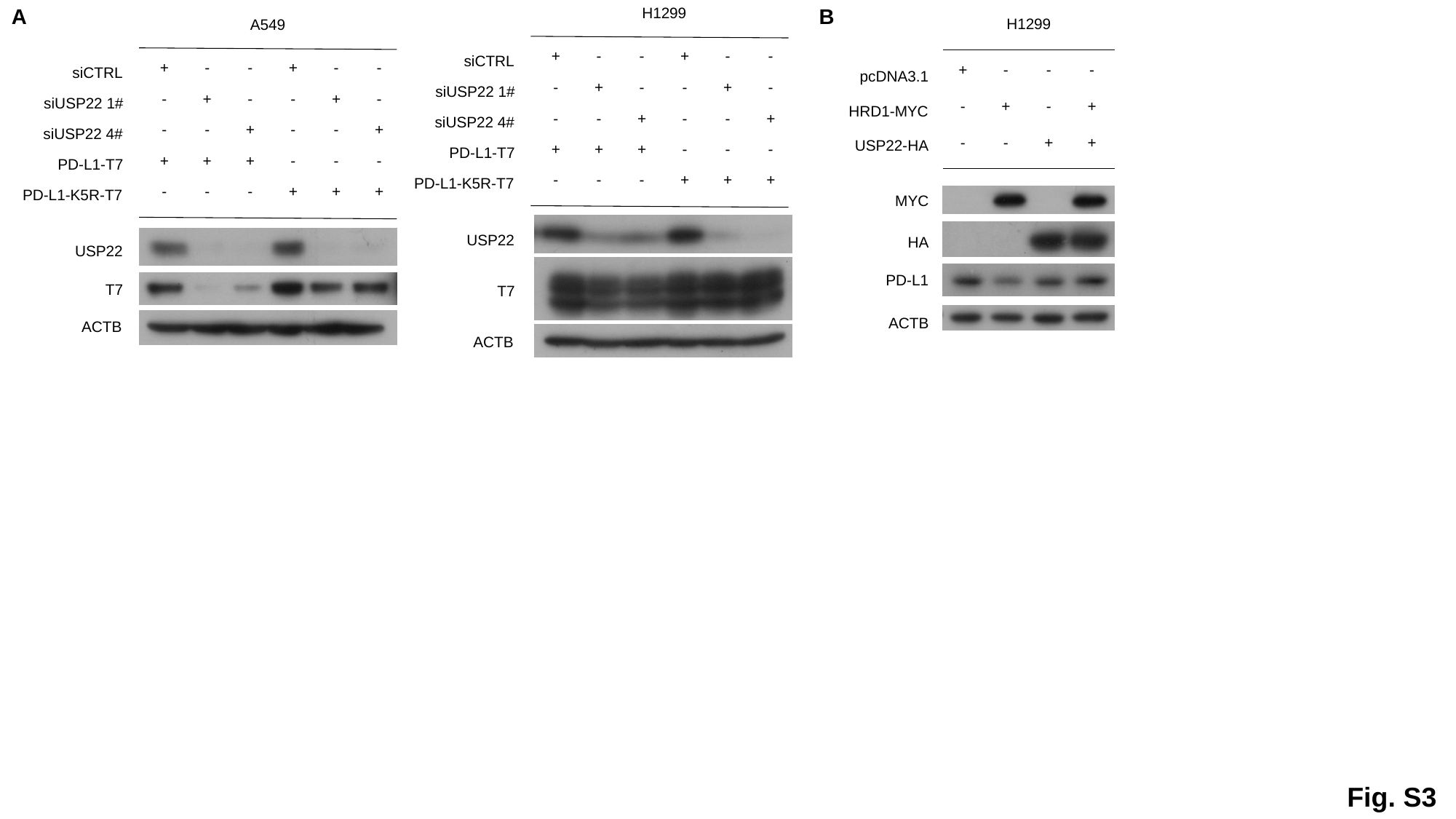

H1299
A
B
 H1299
 A549
| + | - | - | + | - | - |
| --- | --- | --- | --- | --- | --- |
| - | + | - | - | + | - |
| - | - | + | - | - | + |
| + | + | + | - | - | - |
| - | - | - | + | + | + |
 siCTRL
| + | - | - | + | - | - |
| --- | --- | --- | --- | --- | --- |
| - | + | - | - | + | - |
| - | - | + | - | - | + |
| + | + | + | - | - | - |
| - | - | - | + | + | + |
| + | - | - | - |
| --- | --- | --- | --- |
| - | + | - | + |
| - | - | + | + |
 siCTRL
pcDNA3.1
 siUSP22 1#
 siUSP22 1#
HRD1-MYC
 siUSP22 4#
 siUSP22 4#
USP22-HA
PD-L1-T7
PD-L1-T7
PD-L1-K5R-T7
PD-L1-K5R-T7
 MYC
 USP22
 HA
 USP22
 PD-L1
T7
T7
 ACTB
ACTB
ACTB
Fig. S3

## Slide 4
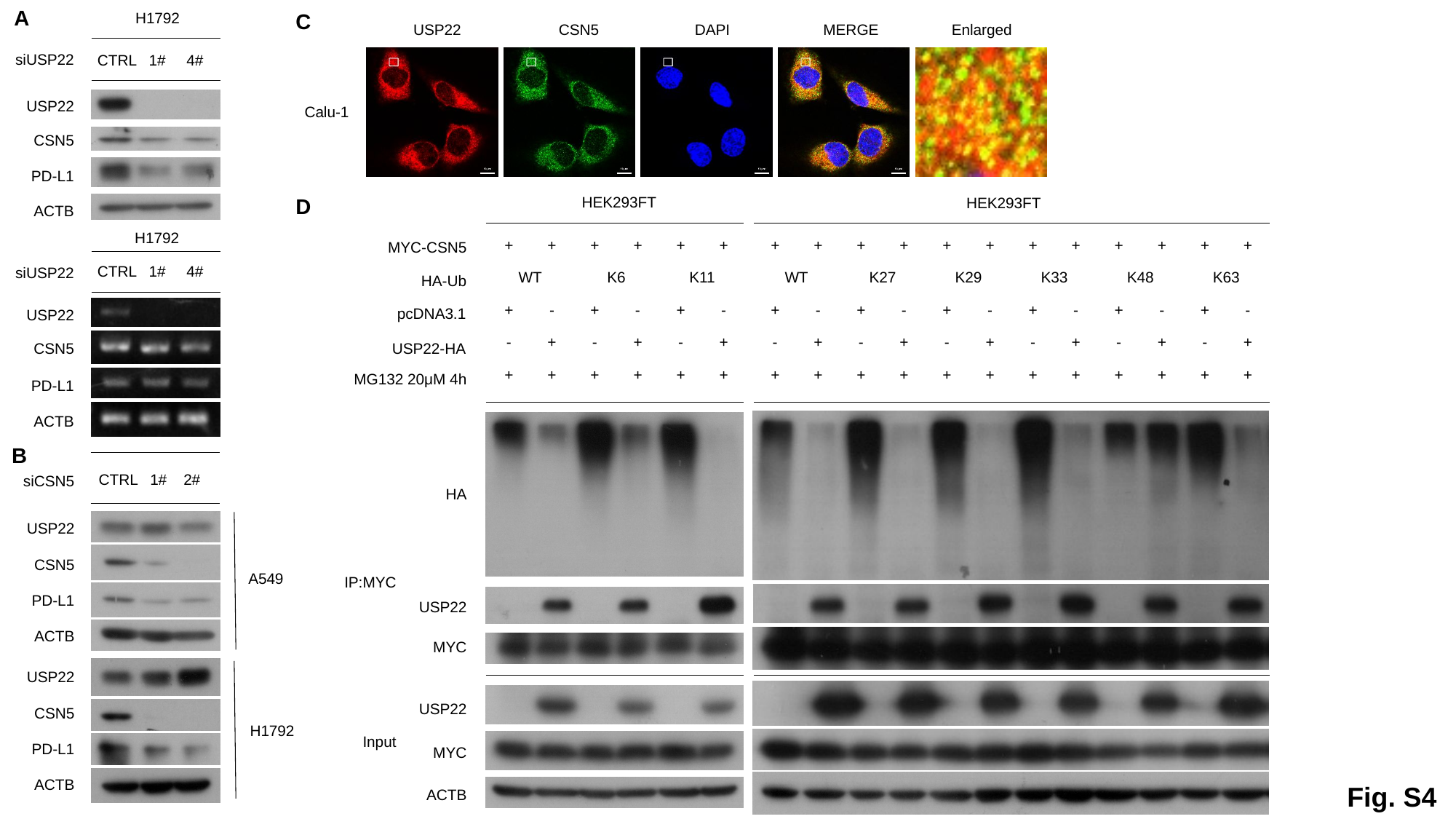

A
 H1792
C
USP22
CSN5
DAPI
MERGE
Enlarged
siUSP22
 CTRL 1# 4#
USP22
 Calu-1
 CSN5
 PD-L1
 HEK293FT
 HEK293FT
D
 ACTB
 H1792
| + | + | + | + | + | + |
| --- | --- | --- | --- | --- | --- |
| WT | | K6 | | K11 | |
| + | - | + | - | + | - |
| - | + | - | + | - | + |
| + | + | + | + | + | + |
| + | + | + | + | + | + | + | + | + | + | + | + | | |
| --- | --- | --- | --- | --- | --- | --- | --- | --- | --- | --- | --- | --- | --- |
| WT | | K27 | | K29 | | K33 | | K48 | | K63 | | | |
| + | - | + | - | + | - | + | - | + | - | + | - | | |
| - | + | - | + | - | + | - | + | - | + | - | + | | |
| + | + | + | + | + | + | + | + | + | + | + | + | | |
MYC-CSN5
 CTRL 1# 4#
siUSP22
HA-Ub
pcDNA3.1
USP22
USP22-HA
 CSN5
MG132 20μM 4h
 PD-L1
 ACTB
B
 CTRL 1# 2#
siCSN5
 HA
USP22
 CSN5
 A549
 IP:MYC
 PD-L1
USP22
 ACTB
MYC
USP22
 USP22
 CSN5
 H1792
Input
 PD-L1
 MYC
 ACTB
Fig. S4
ACTB
