# Supplementary material for: The deubiquitinase USP22 regulates PD-L1 degradation in human cancer cells
Source: Cell Commun Signal. 2020 Jul 14;18:112. doi: 10.1186/s12964-020-00612-y (PMC7362500; doi:10.1186/s12964-020-00612-y)
Supplement: Supplementary file 2 — Additional file 1: Figure S1. USP22 influences PD-L1 protein abundance. Figure S2. USP22 deubiquitinates PD-L1. Figure S3. USP22 deubiquitinates PD-L1. Figure S4. USP22 targets CSN5 for Deubiquitination. [file 12964_2020_612_MOESM2_ESM.zip › legends for Fig. S1-4.docx]

**Fig. S1** USP22 influence PD-L1 protein abundance. (A) MDA-MB-231 and DLD1 cells were transfected with control or USP22 siRNA for 24h and then subjected to western blot analysis. (B) H1299 cells were transfected with PD-L1-FLAG plasmids and increasing amount of USP22 siRNA. PD-L1 and USP22 level were detected by Western blot. (C) H1792 cells were transfected with PD-L1-FLAG plasmids and increasing amount of USP22-HA plasmids. PD-L1 and USP22 level were detected by Western blot. (D) H1792 cells were transfected with control (CTRL) or USP22 siRNA for 24h before RNA was extracted and subjected to reverse transcript PCR (RT-PCR). Protein and mRNA level of USP22 and PD-L1 were assesses using western blot after agarose gel electrophoresis respectively. (E) Exogenous PD-L1 protein level was measured upon USP22 knockdown with or without proteasome inhibitor MG132 in H1792 cells.

**Fig. S2** USP22 deubiquitinates PD-L1. (A) co-immunoprecipitation were carried out using either anti-HA or anti-FLAG antibody in H157 cells. (B) H1299 cells were transfected with indicated constructs. The impact of USP22 overexpression on PD-L1 ubiquitination were tested. (C) HEK293FT cells were transfected with different mutations of Ub. K6, K11 K27, K29, K33, K48, K63 represent only one lysine was natural while all other lysines were replaced with alanines. PD-L1 ubiquitination were explored.

**Fig. S3** USP22 deubiquitinates PD-L1 through intracellular domain. (A) A549, H1299 cells were transfected with control (CTRL) or USP22 siRNA with wild type or mutant PD-L1 plasmids, PD-L1 protein was measured by Western blot. (B) H1299 cells were transfected with pcDNA3.1-USP22-HA and pcDNA3.1-HRD1-MYC plasmid as indicated for 24h, PD-L1 protein was measured by Western blot.

**Fig. S4** USP22 targets CSN5 for Deubiquitination. (A) H1792 cells were transfected with control (CTRL) or USP22 siRNA for 24h before RNA was extracted and subjected to reverse transcript PCR (RT-PCR). (B) A549, H1792 cells were transfected with control (CTRL) or CSN5 siRNA for 24h and then subjected to western blot analysis. (C) Immunofluorescence staining of endogenous USP22 and CSN5. Scale bar, 10µm. (D) HEK293FT cells were transfected with different mutations of Ub. K6, K11 K27, K29, K33, K48, K63 represent only one lysine was natural while all other lysine were mutated to alanine. CSN5 ubiquitination were explored.
