# Supplementary material for: The deubiquitinase USP22 regulates PD-L1 degradation in human cancer cells
Source: Cell Commun Signal. 2020 Jul 14;18:112. doi: 10.1186/s12964-020-00612-y (PMC7362500; doi:10.1186/s12964-020-00612-y)
Supplement: Supplementary file 3 — Additional file 2: Table S1. Correlations between expression levels of USP22 and PD-L1 [file 12964_2020_612_MOESM3_ESM.docx]

Table S1 Correlations between expression levels of USP22 and PD-L1

| **Protein expression** | **PD-L1** | | | **P-value** |
| --- | --- | --- | --- | --- |
|  | **Negative (%)** | **Positive (%)** | **Total (%)** |  |
| **USP22 Negative** | 108 (62.1%) | 27 (40.3%) | 135 (56%) | 0.0023* |
| **USP22 Positive** | 66 (37.9%) | 40 (59.7%) | 106 (44%) |  |
| **Total** | 174 (100%) | 67 (100%) | 241 (100%) |  |

*Chi-square test, statistically significant difference (*P* < 0.05).
